# Exploring the gelation of aqueous cellulose nanocrystals (CNCs)-hydroxyethyl cellulose (HEC) mixtures

**DOI:** 10.1007/s00397-021-01285-1

**Published:** 2021-07-07

**Authors:** Jonathan Stolz, Hale Oguzlu, Zahra Khalili, Yaman Boluk

**Affiliations:** 1 Department of Civil and Environmental Engineering, University of Alberta, Edmonton, AB T6G 2G2 Canada; 2 Present Address: Department of Wood Science, The University of British Columbia, Vancouver, BC V6T 1Z4 Canada

**Keywords:** Cellulose nanocrystals, Hydroxyethyl cellulose, Shear viscosity, Large amplitude oscillatory shear (LAOS), Fourier transform rheology

## Abstract

We investigated the gelation and microstructure of cellulose nanocrystals (CNCs) in nonionic hydroxyethyl cellulose (HEC) solutions. Cellulose nanocrystals (CNCs) with a particle length of 90 nm and width of 8 nm currently produced by acid hydrolysis of wood pulp were used in this study. The microstructures of CNCs/polymer suspensions were investigated by performing linear small amplitude oscillatory shear (SAOS) and nonlinear large amplitude oscillatory shear (LAOS), in addition to constructing CNCs phase diagrams and measuring steady-state shear viscosities. Significant viscosity increases at low shear rates coupled with high shear thinning behaviors were observed in CNCs in HEC solutions above the overlapping concentration of HEC. The physical strength of CNCs/HEC solution gels increased with the increase in CNCs concentration and resembled the weakly crosslinked gels according to the scaling of linear dynamic mechanical experiments. According to LAOS analysis, CNCs/HEC mixtures showed type III behavior with intercycle stress softening, while the samples showed stress stiffening in single cycles.

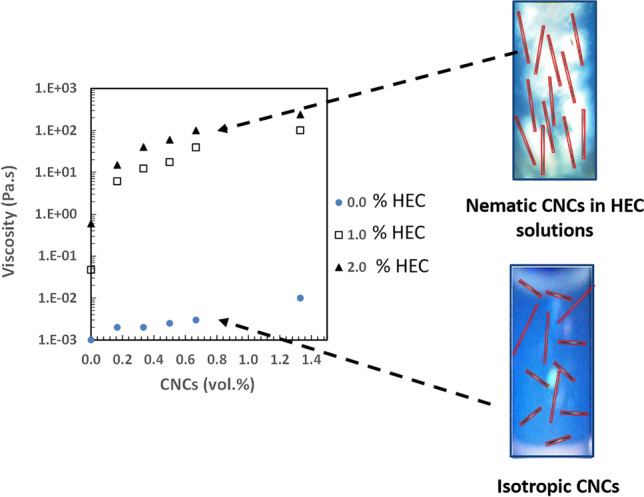

## Introduction

Cellulose nanocrystals (CNCs) whiskers with a particle length of 100–200 nm and a width of 6–10 nm are currently produced in pilot plants by acid hydrolysis of alkaline wood pulp with an expectation of commercialization in the near future (Ngo et al. 1989, O’Connor et al. 2014). They form colloidally stable suspensions in water due to the creation of negatively charged sulfate half ester groups on surfaces (Boluk et al. 1989a, Shafeiei-Sabet et al. 1989, et al.). As a renewable and inherently nontoxic biomaterial, CNCs have attracted scientific and industrial interest over the past two decades, due to their colloidal, mechanical, and optical properties (Habibi et al. 1989, Klemm et al. 1989). The rheological properties of CNCs in aqueous suspensions are also of interest for tailoring special formulations which can be used in oil field applications (Li et al. 1989, Saha et al. 1989), paints and coatings (Koppolu et al. 1989), aircraft anti-icing fluids (Boluk and Zhao 2012), and injectable gels for tissue engineering and thermo-responsive polymers (De France et al. 1989, Way et al. 1989, Yang et al. 1989).

Suspensions of CNCs do not exhibit any thickening in dilute and semi-dilute concentration regimes (typically < 1.0 vol.%) due to the relatively short length of particles (less than 150 nm) unless the CNCs concentration is increased above the concentrated range (Shafiei-Sabet et al. 1989). Colloidal gels can only be formed by creating networks from anisotropic CNCs particles if attractive interactions exist. Alternatively, attractive interactions among CNCs can be realized by screening the electrical double layer repulsions until van der Waals forces remain (Danesh, et al., Moud, et al., Oguzlu et al. 1989b, Phan-Xuan et al. 1989, Shafiei-Sabet et al. 1989). Polymers can also be used in CNC suspensions to create interesting gelling characteristics. Gelling of CNCs in polymer solutions are interest in the applications of injectable gels, 3D printing for tissue engineering and thermo-responsive polymers (De France et al. 1989, Way et al. 1989, Yang et al. 1989). Nevertheless, little attention has been given to the use of semi-dilute CNCs-polymer mixtures to modify rheological properties of aqueous solutions. Our previous study was the first to report the synergetic thickening characteristics of dilute and semi-dilute CNCs dispersions in aqueous polymer solutions such as hydroxyethyl cellulose (HEC) at dilute and semi-dilute concentration regimes where an impressive non-Newtonian thickening behavior (1000 Pa.s) was obtained using a low viscosity CNC (0.002 Pa.s) suspension in a polymer solution (0.8 Pa.s) (Boluk et al. 1989a). Unlike the established means of thickening by entanglement of macromolecular chains, dispersing CNCs in dilute and semi-dilute aqueous polymer solutions provides a better way of controlling shear thinning and elasticity of formulations. In addition, better shear, thermal, and bacterial stabilities can be achieved with much simpler preparation methods.

The stability of colloidal suspensions can be controlled either by adding adsorbing or non-adsorbing polymers (Joanny et al. 1989). Polymer adsorption depends on the polymer–solvent interaction, conformation, molecular weight, and chemical structure of the polymer chain (Fleer et al. 1989). The stability of colloidal suspensions with adsorbing polymers can be described by two mechanisms: (a) adsorption flocculation and (b) steric stabilization (Scheutjens and Fleer 1989). At low polymer concentrations (at low surface coverage), adsorption flocculation is encountered as polymer chains form bridges between particles (Fleer et al. 1993). At higher polymer concentrations (saturated surface coverage), steric stabilization occurs due to repulsive interactions. In the case of polymer depletion, non-adsorbing polymer molecules apply osmotic pressure to particles (Asakura and Oosawa 1989; Vrij 1989). Interestingly, a depletion mechanism can also be seen in the presence of adsorbing polymers (Snowden et al. 1989). After the steric stabilization, with a further increase in polymer concentration, the flocculation of particles occurs by the depletion of excess polymers in the solution. Full coverage of CNCs surfaces turns the excess polymers in solutions into non-adsorbing polymers due to polymer–polymer repulsion in good solvents. CNCs can flocculate and thicken the solutions due to the depletion of excess hydroxyethyl cellulose (HEC) and carboxylmethyl cellulose (CMC) polymers in aqueous solutions (Boluk et al. 1989b, Oguzlu and Boluk 1989).

Peng et al. incorporated CNCs into chitosan, gum arabic, sodium alginate, hydroxypropyl methylcellulose (HPC) and carboxylmethyl cellulose (CMC), polyvinyl alcohol (PVA), and polyethylene glycol (PEG) solutions (Peng et al. 1989). Their results showed that the thickening effect of polymers follows the trend of cationic > anionic > nonionic. They attributed to the excluded volume effects in the case of non-absorbing anionic polymers. In the case of oppositely charged chitosan, the effect was attributed to strong electrostatic interactions. For nonionic polymers such as PVA or PEG, negligible enhancement in the viscosity was observed, and this may be attributed to their more flexible chains. In another study, Lenfant et al. used hydroxyethyl cellulose to induce gelation of CNCs suspensions (Lenfant et al. 1989). This study explained that gelation is by the adsorption of HEC on CNCs and bridging. Hu et al. also reported the adsorption tendencies of hydroxyethyl cellulose (HEC), hydroxypropyl guar (HPG), and locust bean gum (LBG) on CNCs (Hu et al. 1989). However, unlike steric stabilization or bridging, they proposed that the effective volume fraction of dilute CNCs is greatly increased due to the adsorption of polysaccharides and drives CNCs to anisotropic domains and gelation.

HEC polymers are most commonly used as thickening and stabilizing agents in many household and industrial functional formulations. The thickening power of HEC polymers requires the use of either high molecular weight or sufficiently high concentrations. However, high molecular weight HECs become more vulnerable to shear, thermal, and bacterial degradations. On the other hand, high polymer concentrations may penalize shear thinning characteristics at high shear rates. In addition, it is always a challenge to dissolve hydrophilic polymers in aqueous solutions without the formation of lumps in shorter time frames. Therefore, enhancing the rheological behavior of HEC solutions synergistically by the use of a limited amount of CNCs is desirable. Hence, the main objective of this study is to investigate CNCs in HEC solutions under both small amplitude oscillation shear (SAOS) and large amplitude oscillation shear (LAOS) flows in order to gain insight into their microscopic structure. Gel formation due to the addition of CNCs particles in HEC solutions is our interest in exploiting large amplitude oscillatory shear (LAOS) tests in addition to the linear viscoelastic measurements. To our knowledge, only CNCs in salt solutions have been investigated by carrying out LAOS measurements (Moud et al. 1989). This study, therefore, also allows comparisons of viscoelastic deformations due to salt and polymer presences in CNC suspensions under large oscillatory shear deformations.

## Experimental

### Materials

In this study, we did not test any finished products or ingredients on animals, and neither did our suppliers. All of the materials used in this study were based on biomass and carbon neutral. Rod-shaped CNCs particles in dry powder form were received from Innotech Alberta, where they were prepared by sulfuric acid hydrolysis, filtration, and subsequent spray drying (Ngo et al. 1989). A stock suspension with 5% by weight CNC was prepared from powder sample in double distilled deionized water for further use. Weight to volume conversion of NCC suspension concentration was carried out by considering the density of NCC as 1.5 g/cm^3^. CNCs suspensions were prepared by mechanical stirring in deionized water. The mixed solutions were sonicated with Branson ultrasonic cleaner model 1510 (frequency 40 kHz). This CNCs sample was used in a previous study where its dimensions and zeta potential were reported (Boluk et al. 1989b). The effective particle size of the CNC particles was measured as 90 ± 10 nm by dynamic light scattering and taken as the length of particles. The average particle width, determined by image analysis of scanning electron micrographs, was 8 ± 2 nm. The zeta potential of NCC particles was − 51.5 ± 0.8 mV at neutral pH, as determined by a Zetasizer. Sample preparations and particle size analysis methods were reported previously (Oguzlu et al. 1989b).

2-Hydroxyethyl cellulose (HEC) samples were obtained from Sigma Aldrich (St. Louis, MO, USA). According to the supplier’s product specification, HEC720 has a molecular weight (M_W_) of 720 kDa and a molar substitution (MS) of 2.5; HEC250 has a M_W_ of 250 kDa and a MS of 2.0. The viscosity average molecular weights (M_v_) of the HEC samples were determined previously (Boluk et al. 1989b) by measuring intrinsic viscosities ([η]) of aqueous HEC solutions and using [η] = 4.1 × 10^−2^ (M_v_^0.73^) (Clasen and Kulicke 1989). The values of the critical overlap concentrations (c*) of the HEC samples were approximately determined according to Morris et al. by using c * = 4/[η] (Morris et al. 1989). Descriptions of polymers, along with their intrinsic viscosities and critical overlap concentrations (c*), are listed in Table 1.

**Table 1:** Polymers used for solution preparations in experiments

Polymer	Acronym	M.S	[η] (ml/g)	M_v_ (Da)	c* (g/L)
Hydroxyethyl cellulose	HEC250	2.5	296	194,000	13
Hydroxyethyl cellulose	HEC720	2.0	850	819,000	5

### Optical images

Images of CNCs/polymer mixtures were taken as the sample was placed between two crossed, polarized filters in the dark room in which the only source of light is the flash light. The light was beamed through the sample between the filters.

### Rheological measurements

Steady-state shear and dynamic oscillation measurements of CNCs-polymer suspensions were carried out with an AR-G2 rheometer (TA Instruments, USA). Cone-and-plate geometry with a nominal angle of 2°0′22″ and an upper plate of 60 mm in diameter was used for steady-state shear and small amplitude oscillation measurements. A total of 25 mm diameter parallel plate geometry was used with a gap of 500 µm for large amplitude oscillatory shear (LAOS) measurements. The torque resolution was 0.1 μN. In all cases, samples were placed in the test geometry and, once the desired temperature was reached, they were held for 3 min for equilibration before the measurements were started. Steady-state shear measurements were done in a controlled shear rate mode with a ramp time of 10 min. Small amplitude dynamic oscillatory experiments were done in two modes. First, a strain displacement sweep at the frequency of 1 rad/s was carried out, covering both small linear and large strain amplitudes. Then, all of the small amplitude oscillation mode measurements were done within the linear range by sweeping the frequency between 0.1 and 100 rad/s at the 1.0% strain. Large amplitude oscillation tests were conducted as strain sweeps at an oscillation frequency of 1 rad/s. Instantaneous stress, strain, and time data were collected for a period of 120 s at each strain amplitude and were analyzed to determine the coefficients to fit a Fourier series to the stress-time function. Temperature control was established with a ThermoCube200/300/400 (Solid State Cooling Systems Co., USA). Steady-state shear and small amplitude oscillation measurements were conducted at 25 °C, while LAOS measurements were conducted at 20 °C. The wall-slip effect was tested by varying the gap height in the parallel plate geometry while performing amplitude sweep tests.

LAOS analyses were carried out by a sweeping strain amplitude at the angular frequency of 1 rad/s while recording the stress and strain data as a function of time. This stress, strain, and time data was then analyzed to determine the nonlinear viscoelastic coefficients of the material. The software performed a regression fitting of ${\sigma( t;\omega,{\gamma}_{0})}=\gamma_{0\;\; n o d d}{\{{G^\text{'}}_n\left(\omega,\gamma_0\right)\sin\;n\omega t+{G^\text{'}}_n\left(\omega,\gamma_0\right)\cos\;n\omega t\}}$ for *n* from 1 to 9. From the values of *G’*_*n*_ and *G”*_*n*_, the software then calculated the values of *G’*_*M*_, *G’*_*L*_, *η’*_*M*_, *η’*_*L*_, *S*, and *T*. Here, *G’*_*n*_ is nonlinear elastic (storage) modulus from a Fourier series (n = 1, odd), *G”*_*n*_ is the minimum-strain elastic modulus, *G’*_*M*_ is the minimum-strain elastic modulus, *G’*_*L*_ is the largest-strain secant elastic modulus, *η’*_*M*_ is the minimum dynamic viscosity, *η’*_*L*_ is the largest secant dynamic viscosity, *S* = *(G’*_*L*_* − G’*_*M*_) / *G’*_*L*_ and *T* = (*η’*_*L*_* − η’*_*M*_*)* / *η’*_*L*_.These measurements and analyses were performed across a range of *ω* and *γ*_*0*_ to characterize how a material’s rheological response changes under different shearing conditions.

## Results and discussion

### Macroscopic behavior

**Fig. 1:**
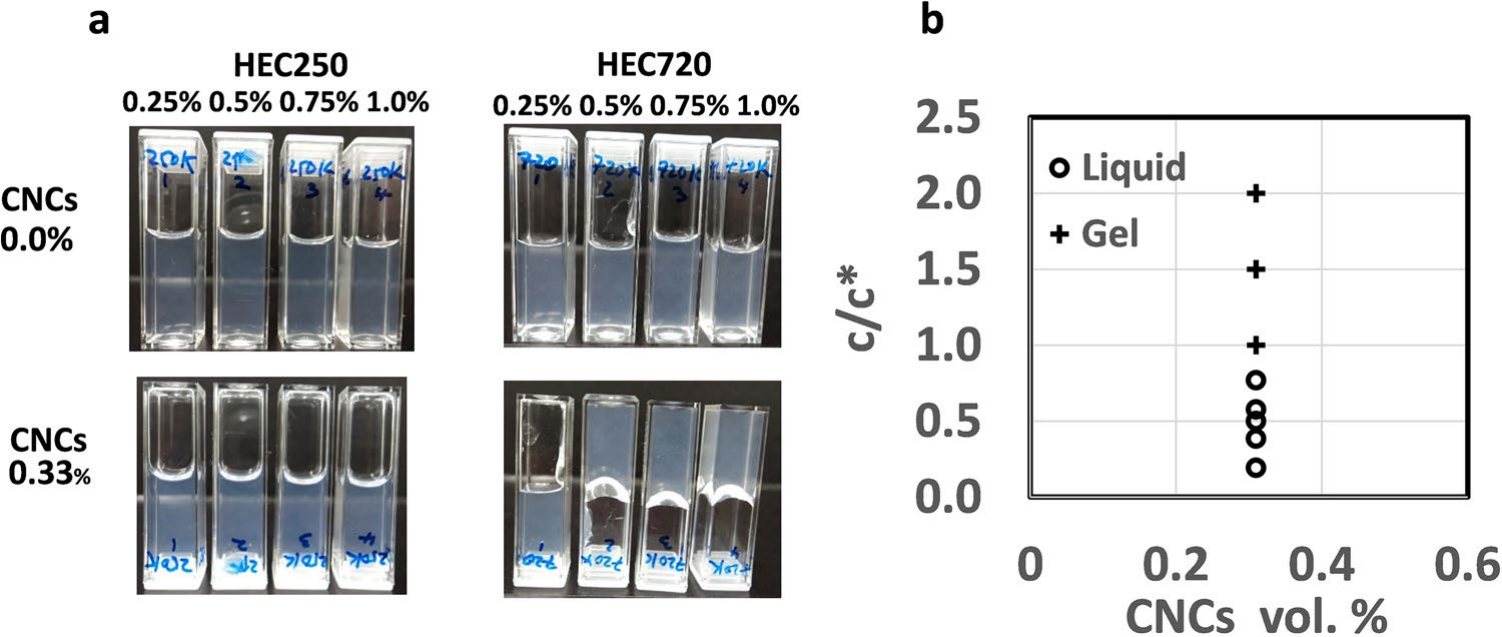
**a** Photographs of hydroxyethyl cellulose solutions with and without 0.33 vol.% CNCs addition; **b** summary of sample inversion results of 0.33 vol.% CNCs in different polymer concentration c/c*

The gelation of CNCs in HEC250 and HEC720polymer solutions was macroscopically investigated by a tube inversion method. Among the samples, only 0.33 vol.% CNCs in 0.5%, 0.75%, and 1.0% of HEC720 polymer solutions stayed suspended on the top of the tubes under their own weight when they were inverted (Fig. 1a). A total of 0.33 vol.% CNCs concentration which is in the dilute regime was chosen to see the maximum synergetic thickening effect in the presence of polymer solutions. Such flow/no flow behavior in polymer solutions was presented as a function of polymer concentration c/c* (Fig. 1b). Gels of CNCs were identified with no flow conditions when the polymer concentration c/c* ≥ *1.0*. Samples with c/c* < *1.0* did not flow instantaneously but in times ranging from 0 to 5 min. Some idea on the yield stress of suspended gels *(τ*_*y*_ = *mg/A* = *50* Pa.) can be obtained based on the weight of a suspended 150 mg gel *(m)* and the contact area of 25 mm^2^ *(A)* of the tube.

### CNCs/HEC720 polymer mixtures

**Fig. 2:**
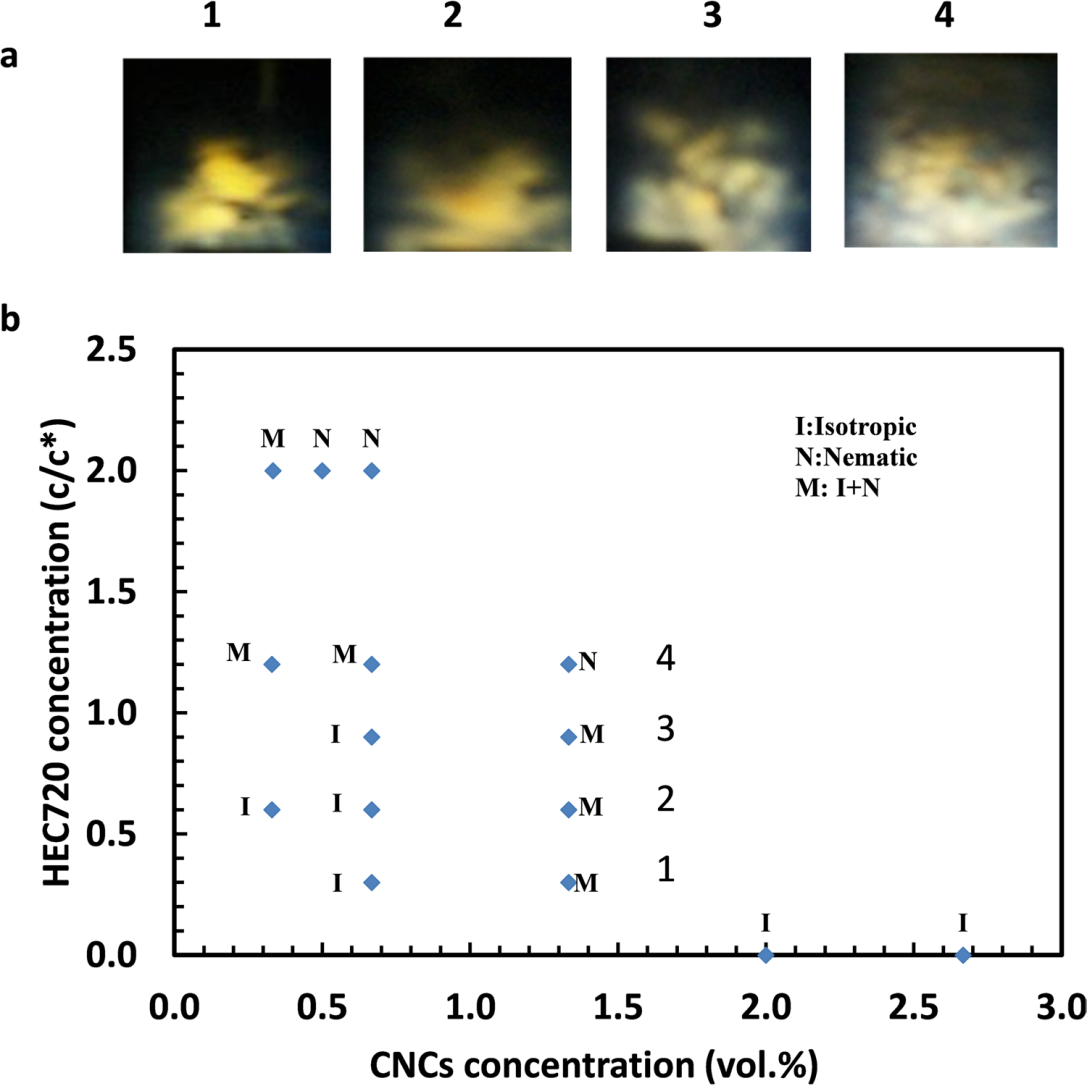
**a** Cross-polarized light transmission results of CNCs/HEC720 mixtures of 1.33 vol.% CNC in 1: 0.15% HEC, 2: 0.3% HEC, 3: 0.45% HEC, and 4: 0.6% HEC, **b** phase diagram for CNCs-HEC720 mixtures (samples 1, 2, 3, and 4 are marked next to data points)

Figure 2a shows the light transmission results of CNCs/HEC720 mixtures of 1.33 vol.% CNC in 1: 0.15% HEC, 2: 0.3% HEC, 3: 0.45% HEC, and 4: 0.6% HEC from cross-polarized filters which were placed before and after samples. Two of those samples (pictures 1 and 2 in Fig. 2) were prepared below the critical overlapping concentration of 0.5% HEC720 (c* = *5 g/L*), one of them (picture 3 in Fig. 2) was around the overlapping concentration and another (picture 4 in Fig. 2) was at a higher than critical overlapping concentration, by selecting HEC720 concentrations of 0.15%, 0.30%, 0.45%, and 0.60%. Obstruction of the light by the suspension of isotropic portions between cross-polarized filters generated dark images. On the contrary, in the case of non-isotropic and oriented particles, the light reflected from the ordered CNC suspensions with different angles resulting in bright areas on images. Hence, the cloudy-white part of the pictures indicates the liquid crystal regions. Only a small part turned cloudy-white in picture 1, which had only 0.15% HRC720. That area increased in picture 2 by increasing the polymer concentration from 0.15 to 0.30%. The bright parts showing the presence of liquid crystals were expanded in sample 3 by increasing the HEC720 concentration to 0.45%. Nevertheless, the rest of the polymer-colloid solution remained dark. Therefore, the mixtures in pictures 1, 2, and 3 were considered mixtures of isotropic + nematic (I + N) phases. The amount of liquid crystals in the suspension increased and occupied almost the whole volume in picture 4 when the polymer concentration was increased to 0.60%. Hence, the sample in picture 4 was called a “Nematic suspension.” The rest of the data points were collected in a similar fashion by varying the CNCs and HEC720 concentrations and the phase diagram of CNCs/HEC720s system is plotted in Fig. 2b. The data points represent samples with isotropic (I), nematic (N), and isotropic + nematic (I + N) mixtures. There was no phase separation due to the formation of nematic structures. A total of 0.33% of CNCs in 0.30% (c/c* = *0.60*) concentration of HEC720 were isotropic and in a liquid state. The CNCs stayed in isotropic + nematic (I + N) mixtures and formed gel in the presence of HECs above the concentration of 0.60% (c/c* = *1.2*). CNCs with 1.5 vol.% concentration stayed in mixtures of I + N at the lowest HEC720 concentration of c/c* = 0.30 and in gel form. All of the CNCs observed as mixed I + M states were also in gel form. CNCs without the addition of any HECs were isotropic and in a liquid state at 2.0% and 2.7% concentrations. Depending on the length to diameter ratio (L/D), CNCs suspensions must be higher than 5 vol.% to exhibit the nematic structures (Oguzlu et al. 1989a). The phase diagram plotted in Fig. 2 agrees with Hu et al.’s Fig. 6, captioned “Minimum CNC volume percent for gelation (inversion test) as a function of the polysaccharide concentration” (Hu, Cranston, Ng and Pelton).

### Steady-state shear flow

**Fig. 3:**
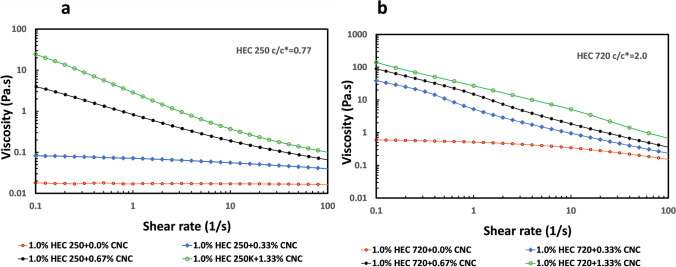
Steady shear rate viscosities of 0.33% and 0.67% CNCs as a function of shear rate in: **a** 1.0% HEC250; **b** 1.0% HEC720

Figure 3 shows plots of steady-state shear viscosity as a function of the applied shear rate of 1.0% (10 g/L) HEC250 and HEC720 polymer solutions at 0.33%, 0.67%, and 1.33% CNC concentrations. The polymer concentration of 1.0% was below the critical overlapping concentration, c^*^ of HEC250 (c^*^ ~ *13.0 g/L*), but above the c^*^ of the HEC720 (c^*^ ~ *5 g/L*). In the case of CNCs, the transition from dilute to semi-dilute concentration occurs at a volume fraction of *ϕ* ≤ *(π/4)(d/L)*^*2*^ where d is the diameter and L is the length of the CNC rods. This equation suggests that CNCs suspension at 0.33% is within the dilute regime, 0.67% is just above the dilute concentration range where particles barely contact each other and 1.33% is semi-dilute. The viscosity of 0.33%, 0.67%, and 1.33% CNCs suspension is 0.002, 0.003, and 0.01 Pa.s respectively at 10^−2^ shear rate without any polymer addition (Oguzlu et al. 1989a). As expected, the HEC250 polymer solution at c/c* = *0.77* showed Newtonian as opposed to non-Newtonian behavior of HEC720 at c/c* = *2.0*. Additions of CNCs above their dilute concentration regime (> 0.66%) increased the solution viscosities of HEC solutions at low shear rates showing strong shear thinning behavior. Viscosity enhancing character of CNCs in HEC solutions is reported in other studies (Boluk et al. 1989b, Hu et al. 1989).

**Fig. 4:**
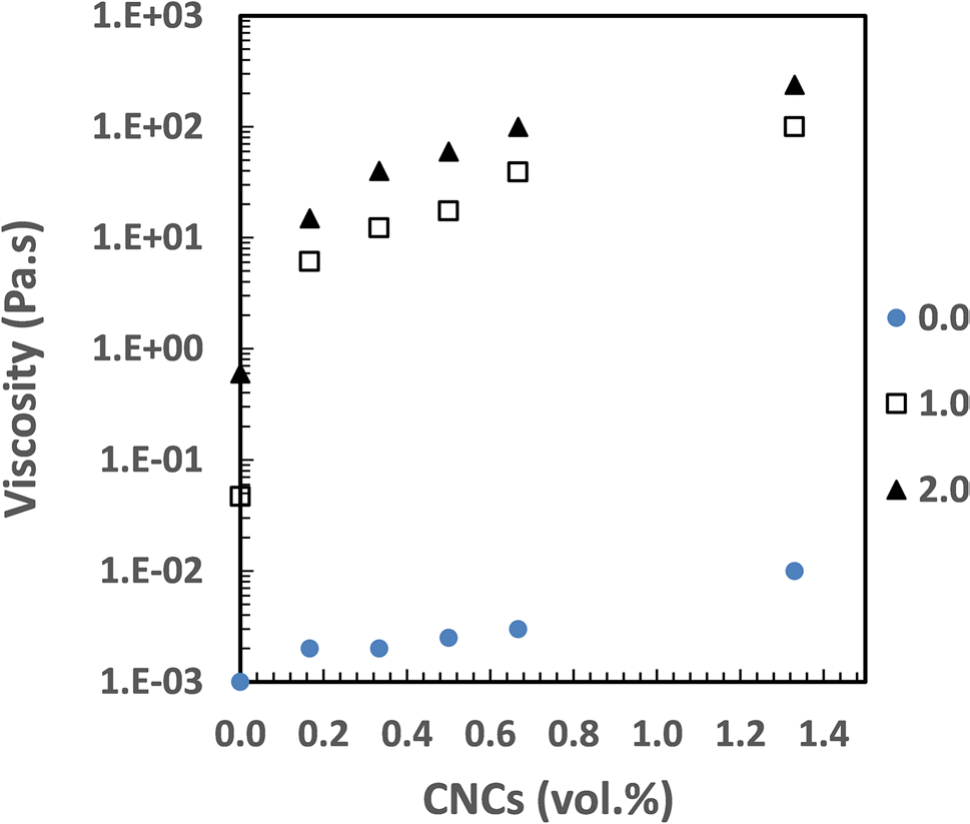
Shear viscosity (at 0.1 s^−1^) vs. CNCs concentration in HEC720 polymer solutions. Legend symbols on the right side show the c/c* of HEC720 polymer solution

Figure 4 shows shear viscosities as a function of CNCs concentration in various HEC720 solutions. The addition of CNCs, even at its diluted regime (< 0.4 vol.%), significantly increased the low shear rate (0.1 s^−1^) viscosity of HEC720 at 0.5% (c/c* = *1.0*) and 1.0% (c/c* = *2.0*) concentrations. This kind of strong shear viscosity enhancement by CNCs at low shear rates has many potential industrial uses such as in wall paints and other sprayable functional fluids, where it is desirable that they do not flow too quickly under gravity but are easy to handle and apply.

### Frequency sweeps at a low amplitude strain

**Fig. 5:**
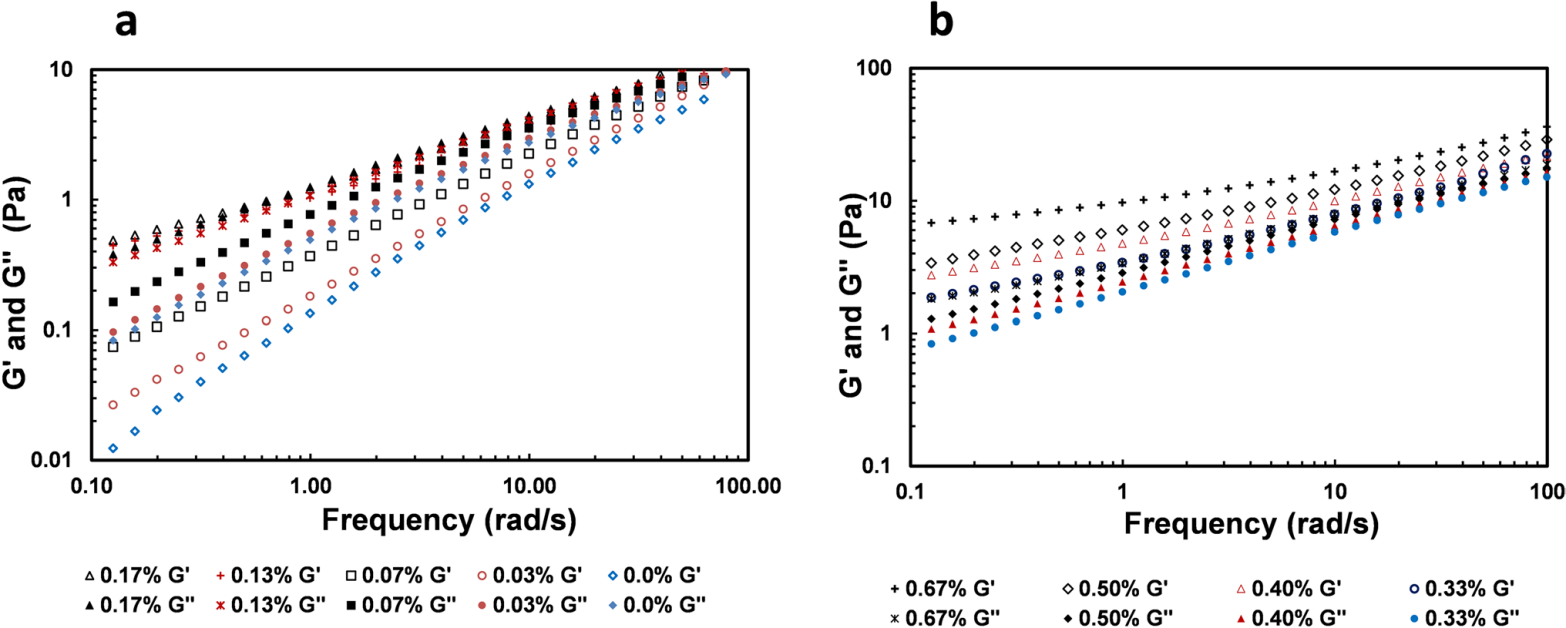
G’(ω) and G”(ω) of 1.0% (c/c* = 2.0) HEC720 solution with the presence of CNCs. CNC concentrations (vol.%) are **a** from 0.0 to 0.17%, **b** from 0.33 to 0.67%

Linear viscoelastic properties of CNCs/HEC720 mixtures were measured by varying the CNCs concentrations from 0.0 to 0.67% in 1.0% HEC720 solutions to investigate the contributions of CNCs particles (Fig. 5). Newtonian viscosity, *η*_*0*_ of 1.0% HEC720 solution, was calculated as 0.58 Pa.s from *G”* = *η*_*0*_*ω* at low frequencies which agreed with the shear viscosity of 0.60 Pa.s measured at 0.1 s^−1^ steady-state shear rate. A total of 1.0% HEC720 polymer solution without any CNC presence (0.0%) has a loss modulus (*G”*) ten times higher than its elastic modulus (*G’*) at 0.1 rad/s angular frequency. *G”(ω)* of the polymer solution was still two times higher than *G’(ω)* at 100 rad/s angular frequency. With the absence of the crossover between *G*’ and *G”*, the 1.0% HEC720 solution was viscous with a weak viscoelasticity. The storage modulus and loss modulus of 1.0% HEC720 had the power-law relation of *G′∼ω*^*1.0*^, *G″∼ω*^*0.8*^, respectively. The addition of CNC particles into the HEC720 solution gradually increased both the elastic and the loss moduli. The decrease in the slope of G’(ω) was higher than the decrease in the slope of *G”(ω)* with an increase in CNCs concentration. Nevertheless, the elastic moduli of 0.03%, 0.07%, 0.13%, and 0.17% CNCs suspensions were still lower than their corresponding loss moduli (*G’* < *G”*) at all measured frequencies (Fig. 5a). With the CNCs concentration of 0.20%, G’ crosses over G” at ω = 31 rad/s. The increase in elasticity with the increase in CNCs volume fractions becomes substantial above 0.20%. The elastic modulus of 0.27% (not shown on the plot) almost overlapped with its loss modulus, while 0.33%, 0.40%, 0.50%, and 0.67% CNCs suspensions in 1.0% HEC solutions had a higher elastic modulus than their loss moduli (G’ > G”) at all measured frequencies (Fig. 5b). Those plots also showed a tendency towards an elastic plateau modulus at low frequency ranges as CNCs concentration increased. Nevertheless, these compositions had lower level of gel formation than high aspect ratio molecular nanofibers (Raghavan and Douglas 1989). The observed rheological behavior indicates that CNCs particles formed a flocculated and networked structure as the concentration increased. At the highest measured CNC volume fraction, φ = 0.67%, the suspension was clearly elastic and *G’(ω)* was 1.7 times higher than *G”(ω)* at the angular frequency of 100 rad/s.

*G’* and *G”* of 0.67% CNCs suspension were found to have weak power-law relations with frequency (*G’∼ω*^*n*^* and G”∼ω*^*m*^), while having exponents of n = 0.24 + 0.1 and m = 0.36 + 0.1. The weak power-law frequency dependencies of *G’* and *G”* are characteristic features of gel structures. The gelation of CNCs suspensions in HEC solution can be explained by the appearance of microstructural formations due to the flocculated structures. Winter and Chambon showed a similar gelling behavior due to the crosslinking of polymers by analyzing linear viscoelasticity (Winter and Chambon 1989). The higher “m” value of *G”* ~ *ω*^*m*^ indicated the structural relaxation of CNCs-HEC720 gels at low frequencies (longer times), while the lower “*n*” value than “*m*” was attributed to the dominant contribution of inter-cluster links between CNCs. Similar behaviors were also observed in other depleted colloidal gels (Laurati et al. 1989).

**Fig. 6:**
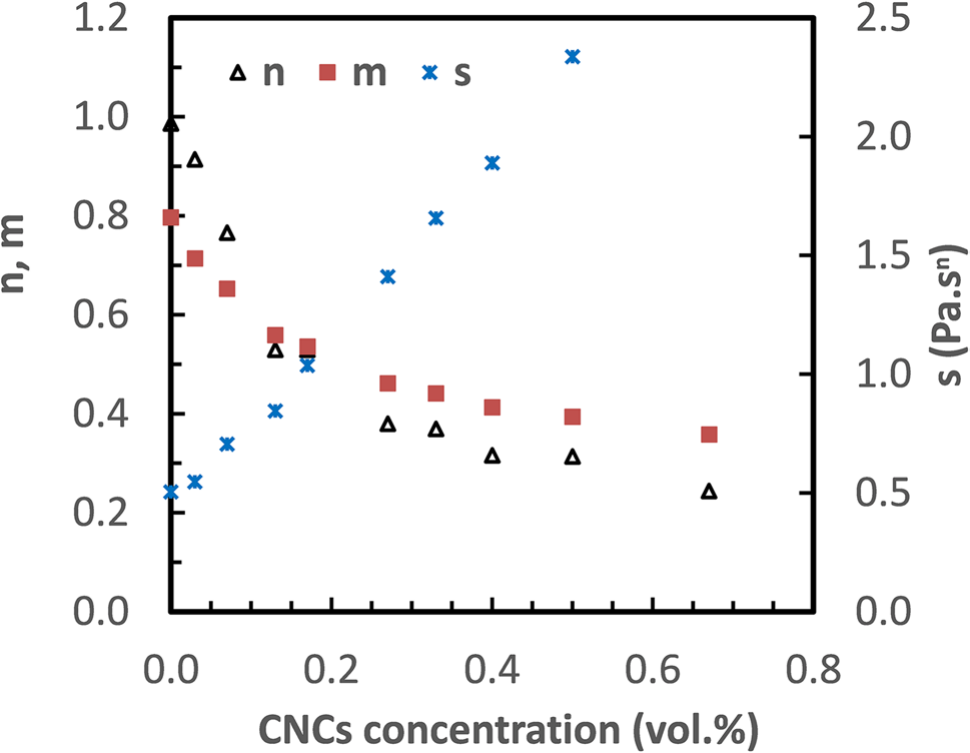
Plots of the elastic and loss power-law constants *n*, *m*, and gel strength parameter *s* for CNCs in 1.0 HEC720

An intermediate level of gel formation can be described with the shear relaxation modulus by a power-law in time *G(t)* = *st*^*−n*^ at the gel point, where *s* is the gel strength with a dimension of Pas^n^ and *n* is the relaxation exponent (Chambon and Winter 1989, Izuka et al. 1989). The same scaling can be applied in dynamic mechanical experiments where the storage modulus, *G’* and the loss modulus, *G”* at the gel point are given as follows (Lu et al. 1989a, b, Nyström et al. 1989)

1 $${G}^{\text{'}}\left(\omega \right)=\frac{{G}^{"}(\omega )}{{t}{a}{n}(\delta )}=s{\omega }^{n}{\Gamma }\left(1-n\right){c}{o}{s}(\frac{n\pi }{2})$$

where ${\Gamma }\left(1-n\right)$ is the Legendre gamma function. Power-law constants of *n (G’∼ω*^*n*^*)* and *m (G”∼ω*^*m*^*)* of CNCs in HEC720 polymer solutions were calculated from the dynamic mechanical data in Fig. 5. The gel strength constant at various CNCs concentrations was calculated by using Eq. 1. Figure 6 shows the plots of calculated *n*, *m*, and *s* values vs. CNCs concentrations. Both *n* and *m* values decreased with the CNCs concentration; however, the decrease in *n* was higher than *m* and, above the CNCs concentration of 20%, *n* was smaller than *m*. The smaller values of *n* above 30% of CNCs indicate highly elastic gel formations (Gao and Nishinari 1989, Kjøniksen et al. 1989, Nordby et al. 1989). The gel strength *s* increased while n decreased with the increase of CNCs concentration which suggests the physical strength of the gel network at the gel point is similar to crosslinked gels (Kjøniksen and Nyström 1989). Interestingly, the *s* values—in other words, the gel strength of CNCs in nonionic HEC solution—reported here were stronger than the cationic polyelectrolyte quaternized hydroxyethylcellulose ethoxylate (QHEC) solution, reported previously (Lu et al. 1989a, b).

The network of CNCs particles contributed significantly to the viscoelastic behavior of suspensions while the continuous polymer solution was fluid. The *G’(ω)* and *G”(ω)* curves of CNCs in HEC solution above 0.2% were similar but they shifted as the CNC concentration increased. For carbon black particles suspended in Newtonian base stock oil, Trappe and Weitz proposed that the fluid component contributes only the viscous loss component while the particulate network is elastic. A similar argument was also tested later for a particulate silica network in non-Newtonian carboxymethyl cellulose and xanthan gum solutions. The existence of a crossover frequency (*ω*_*c*_) at very high frequencies indicated that the elastic nature of the CNCs network and the viscous nature of the HEC720 polymer solution are practically independent of each other. Therefore, the viscoelastic behavior of CNCs in 1.0% HEC720 solution can be scaled onto a single master curve by shifting their curves horizontally and vertically.

### Large amplitude oscillatory shear

**Fig. 7:**
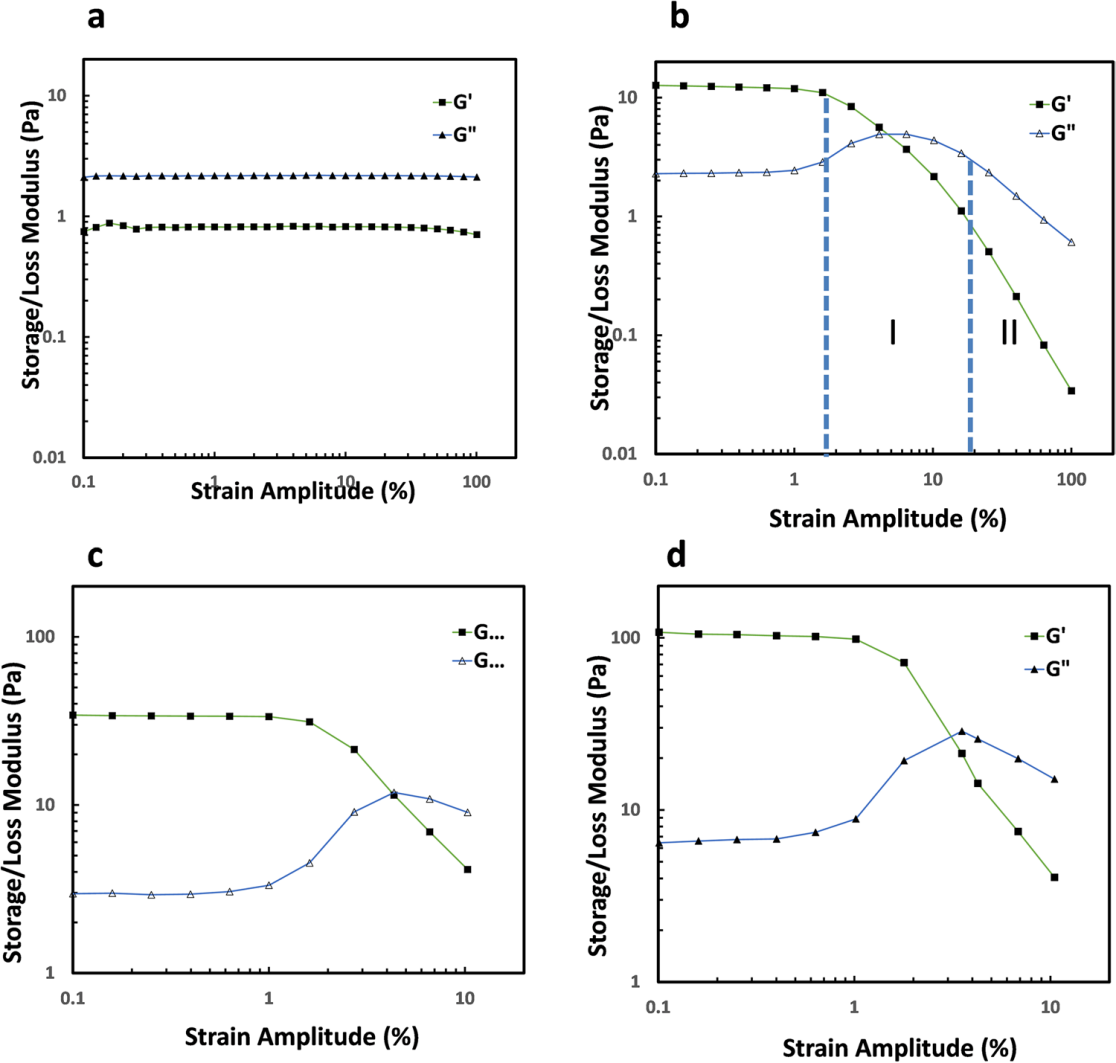
Amplitude sweeps of 1.0 HEC720 solutions at the frequency of 1.0 rad/s with **a** 0.0% CNCs, **b** 0.33% CNCs, **c** 0.67% CNCs, **d** 1.33% CNCs (data points are connected to guide the eyes)

Gel structures of CNCs in HEC720 solutions were further investigated by running amplitude sweeps at 1.0 rad/s oscillation frequency (Fig. 7). The polymer solution of 1.0% HEC720 without the addition of CNCs particles was a viscous fluid with *G”* > *G’* and both storage and loss moduli were independent of oscillatory strain up to 100%. In contrast, CNCs in HEC720 solutions exhibited *G’* > *G”* while G’ and *G”* were constant only up to a certain strain amplitude because of their gel-like behavior. In those mixtures, the linear regime was present only up to the strain amplitude of around 1 rad/s. 0.33%, 0.67%, and 1.33% CNCs and had a *G’* value of 13.4 Pa, 37.8 Pa, and 108 Pa respectively within the linear range. Above 1 rad/s, G’ decreased logarithmically from its linear plateau values due to the stress softening referred to as the “Payne Effect” (Payne 1989a, b). As discussed by Wu et al. (Moud et al. 1989) and Moud et al. (Moud et al. 1989), it is believed that the linear elastic behavior of CNCs/HEC700 mixtures disappeared when the weakest links among CNCs particles due to the van der Waals interactions were broken.

**Fig. 8:**
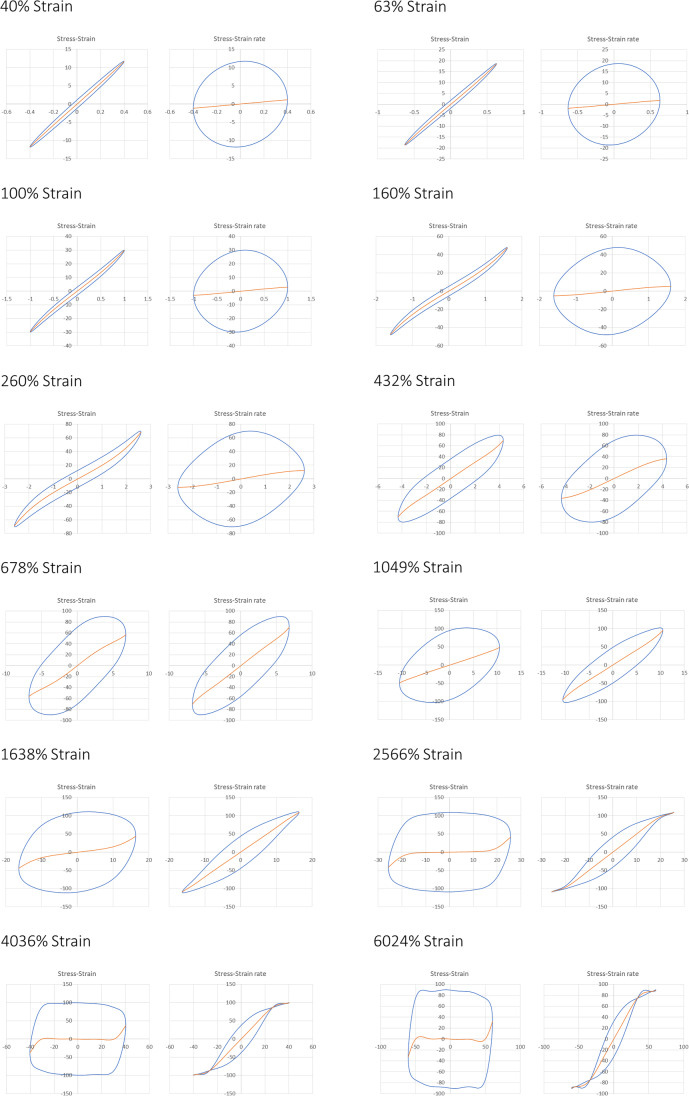
Lissajoux-Bowditch plots of 0.66% CNC + 1.0% HEC700 polymer solutions. The strain amplitude of each pair of stress–strain and stress–strain rate plots are shown on the right side of the plot

In the case of G”, there was an initial increase up to a maximum point from its linear strain region value and then a decrease as the amplitude of the strain was increased. This type of overshoot is classified as type III behavior. The span of overshoot in a type III gel can be discussed in two regions (Fig. 8b). Within region I, flocculated CNCs particles in HEC solutions resisted the deformation up to a certain point where G” reached the maximum point. Above the critical strain in region II, where CNCs particles aligned with the shear field and G” decreased, the complex structure was destroyed. The portion of deformation energy which is lost by internal friction during the shearing action contributed to the loss modulus G”. Deformation energy is lost because of free moving, flocculated CNCs that are no longer integrated within microstructures. The magnitude of overshoot in G” increased as the CNCs concentration in the polymer solution was increased (from b to d in Fig. 7). Nevertheless, the maximum point of *G”* did not change significanly among samples. The overshoot of *G”* is also due to the “Payne Effect” which has been observed in other yield stress fluids and associated with bond breaking among load bearing elements (Allegra et al. 1989; Raghavan and Khan 1989) . According to the network model composed of segments and junctions as discussed by Hyun et al., type III behavior occurs when both creation and destruction rates are positive but the creation rate is smaller than the destruction rate (Hyun et al. 1989). A similar type III behavior, but with weaker *G”* overshoots values, was observed in cellulose nanocrystals suspensions in the presence of electrolytes (Danesh et al. 1989, Moud et al. 1989). In the case of CNCs in aqueous polymer solutions, Chen et al. reported a strain overshoot of G” with 2 wt.% CNCs in 10% PVA (Mw = 43 kDa) solution but not in 2% CNCs in 1.0% CMC (Mw = 260 kDa) solution (Chen et al. 1989). It is worth noting that the stress dependencies of moduli are also strongly related to the surface properties of plates. The crossover of *G’* and maximum of *G”* obtained here using smooth plates might be lower than the actual values due to the wall slip. According to Yang and Yu, the wall-slip effect becomes weak above the yield stress in a liquid-like regime at higher strain amplitudes and *G”* shows much stronger variation than G’ when the stress amplitude is just above the critical slip stress (Yang and Yu 1989).

For a qualitative evalution of large amplitude oscillatory shear tests, the closed loop Lissajoux-Bowditch plots of 0.66% CNC + 1.0% HEC720 polymer solutions were prepared (Fig. 8). A predominantly linear viscoelastic regime was observed at low strains of 40% and 63%. For strains above 100%, the material showed nonlinear responses. Above 2566% strain amplitude, the stress vs. strain curves distorted to rectangular shapes and they were shear thinning. The behavior of those CNCs/HEC700 solutions resembled aqueous solutions of anionic, stiff, semi-rigid xanthan gum polysaccharide (Hyun et al. 1989).

**Fig. 9:**
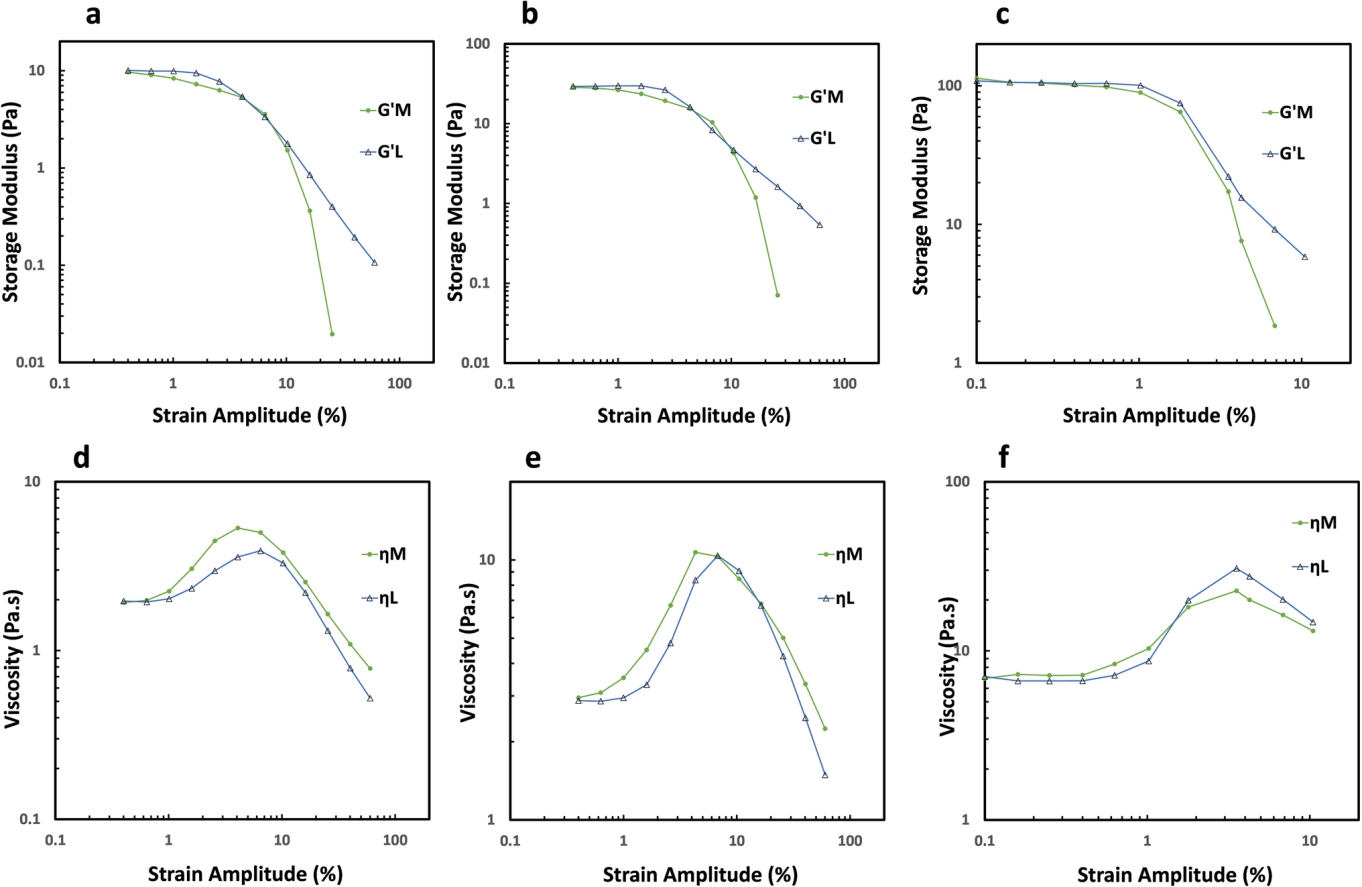
Intracyle viscoelastic behavior of CNCs suspensions in 1.0 HEC720 solution at the frequency of 1.0 rad/s. Local viscoelactic moduli G_M_’ and G_L_’ of **a** 0.33% CNCs, **b** 0.67% CNCs, **c** 1.33% CNCs. Local dynamic viscosities η_M_’ and η_L_’ of **d** 0.33% CNCs, **e** 0.67% CNCs, **f** 1.33% CNCs (data points are connected to guide the eyes)

The nonlinear viscoelastic behavior of CNCs-polymer suspensions by large amplitude oscillatory shear (LAOS) experiments was further characterized by Fourier transform rheology and Chebyshev stress decompositions with the procedure laid out by Ewoldt et al. (Ewoldt et al. 1989) and reviewed by Saengow and Giacomin (Saengow and Giacomin 1989). This procedure allows us to investigate the local behavior of CNCs/HEC720 mixtures in a given (intra) cycle by using the minimum (tangent) modulus $G_M^\text{'}=\left.\frac{d\sigma}{d\gamma}\right|\gamma=0$ and large strain (secant) modulus $G_L^\text{'}=\left.\frac\sigma\gamma\right|\gamma=\pm\gamma_0$. The three CNCs mixtures demonstrated qualitatively very similar behavior with decreases in *G*_*M*_*’* which became bigger than *G*_*L*_*’* at higher strain amplitudes but converged to *G’* at the linear range (Fig. 9a–c). The higher value of elasticity at high strains, *G*_*L*_*’* relative to elasticity at low strains *G*_*M*_*’*, shows that CNCs suspensions are strain stiffening within a given cycle, referred to as “intracycle stiffening.” The intercycle data from the strain sweep is the most reliable indicator showing stress softening in CNCs samples (Fig. 7b–d). Nevertheless, intracycle behavior (Fig. 9a–c) showed the local behavior of CNCs particles within a given cycle. Similar conclusions for inter and intracycle behaviors were also reached by Mermet-Guyennet (Mermet-Guyennet et al. 1989) and Ewoldt and Bharadwaj (Ewoldt and Bharadwaj 1989).

**Fig. 10:**
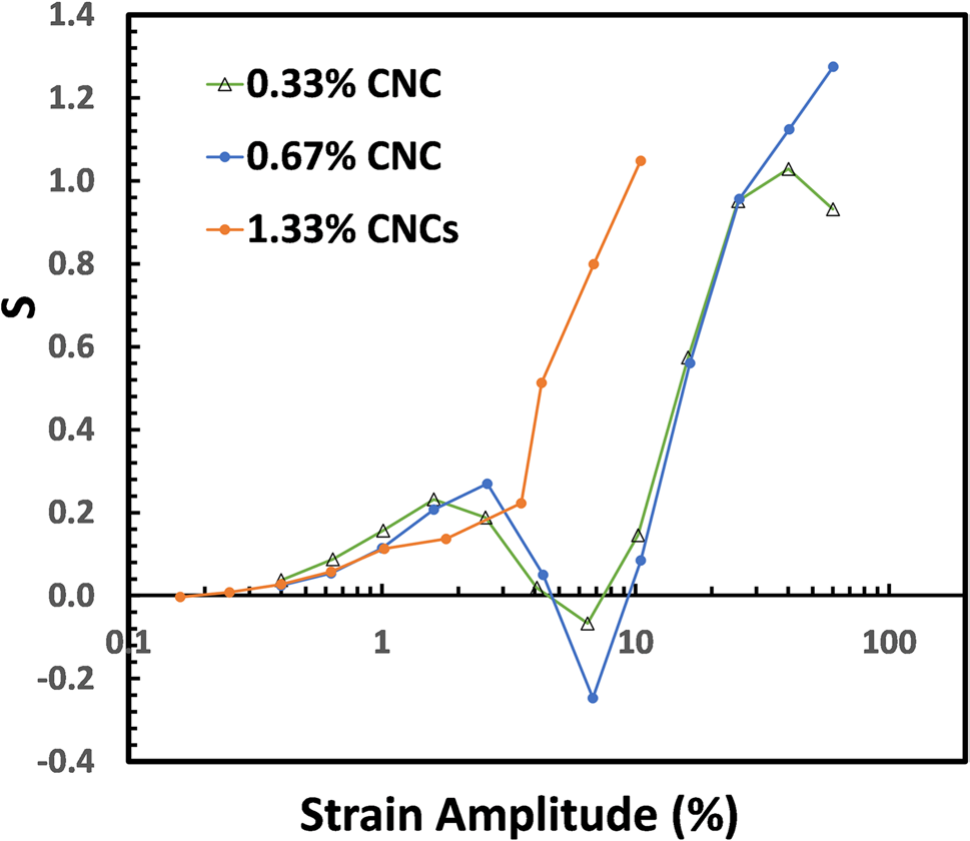
Amplitude dependency of intracycle stiffening index (*S*)

All of the dissipated energy per cycle per unit volume in large amplitude oscillatory shear is still represented by first harmonics loss modulus *G”* (Giacomin et al. 1989). However, *G*” cannot differentiate any local changes in the coefficient of viscous losses between the lowest and highest instantaneous shear rates during a cycle. Ewoldt et al. defined minimum-rate dynamic viscosity $\eta_M^\text{'}=\left.\frac{d\sigma}{d\dot\gamma}\right|\dot\gamma=0$ and large-rate dynamic viscosity $\eta_L^\text{'}=\left.\frac\sigma{\dot\gamma}\right|\dot\gamma=\pm\dot{\gamma_0}$ in order to calculate the viscous response similar to viscoelastic moduli (Ewoldt et al. 1989). The intracycle viscous behavior of CNCs/HEC700 polymer mixture solutions is shown in Fig. 10d–f; *η*_*L*_*’* and *η*_*M*_*’*; they were not equal but behaved in tandem.

The nonlinearity of viscoelastic measures *G*_*M*_*’* and *G*_*L*_*’* is expressed with the dimensionless index of nonlinearity $S\left(=\frac{G_L^\text{'}-G_M^\text{'}}{G_L^\text{'}}\right)$ (Ewoldt et al. 1989). Figure 10 shows the intracycle stiffening of CNCs/HEC720 mixtures. In the 0.33% and 0.66% samples, there was a dip within the strain amplitude of 6%. Nevertheless, intracycle stiffening increased with CNCs concentration. Since $T\left(=\frac{\eta_L^\text{'}-\eta_M^\text{'}}{\eta_L^\text{'}}\right)$ values were small, they were not used to characterize the intracycle shear thinning/thickening behavior of samples.

LAOS experiments displayed evidence of both shear thinning and thickening behavior of dilute and semi-dilute CNCs suspensions in 1.0% HEC solutions. The transition from intracycle shear thickening to intracycle shear thinning behavior shifted to higher strain amplitudes by increasing the frequency of oscillation.

## Conclusions

Even at the lowest concentration of 0.33vol.%, CNCs formed anisotropic structures and gels by the addition of HEC720 polymer just above its overlapping concentration. This resulted in strong shear thinning non-Newtonian behavior by adding CNCs at very low dosages to semi-dilute HEC solutions. Both dynamic viscoelastic measurements within the linear range at small amplitude oscillation shear (SAOS) and nonlinear large amplitude oscillation shear (LAOS) measurements showed the presence of microstructures in CNCs/HEC720 solutions. The physical strength of CNCs/HEC720 solution gels increased with an increase in CNCs concentration and resembled the weakly crosslinked gels according to the scaling of linear dynamic mechanical experiments following the power-law in time shear relaxation modulus, *G(t)* = *st*^*−n*^. According to LAOS analysis, CNCs/HEC720 mixtures showed type III behavior with intercycle stress softening while samples showed stress stiffening in single cycles. The shape of the Lissajous-Bowditch plots distorted to rectangular shapes upon increasing the strain amplitude, which showed the shear thinning character with a yield stress.
